# EMAGE mouse embryo spatial gene expression database: 2014 update

**DOI:** 10.1093/nar/gkt1155

**Published:** 2013-11-20

**Authors:** Lorna Richardson, Shanmugasundaram Venkataraman, Peter Stevenson, Yiya Yang, Julie Moss, Liz Graham, Nicholas Burton, Bill Hill, Jianguo Rao, Richard A. Baldock, Chris Armit

**Affiliations:** MRC Human Genetics Unit, Institute of Genetics and Molecular Medicine, University of Edinburgh, Western General Hospital EH4 2XU, UK

## Abstract

EMAGE (http://www.emouseatlas.org/emage/) is a freely available database of *in situ* gene expression patterns that allows users to perform online queries of mouse developmental gene expression. EMAGE is unique in providing both text-based descriptions of gene expression plus spatial maps of gene expression patterns. This mapping allows spatial queries to be accomplished alongside more traditional text-based queries. Here, we describe our recent progress in spatial mapping and data integration. EMAGE has developed a method of spatially mapping 3D embryo images captured using optical projection tomography, and through the use of an IIP3D viewer allows users to view arbitrary sections of raw and mapped 3D image data in the context of a web browser. EMAGE now includes enhancer data, and we have spatially mapped images from a comprehensive screen of transgenic reporter mice that detail the expression of mouse non-coding genomic DNA fragments with enhancer activity. We have integrated the eMouseAtlas anatomical atlas and the EMAGE database so that a user of the atlas can query the EMAGE database easily. In addition, we have extended the atlas framework to enable EMAGE to spatially cross-index EMBRYS whole mount *in situ* hybridization data. We additionally report on recent developments to the EMAGE web interface, including new query and analysis capabilities.

## INTRODUCTION

The process of mammalian development is highly complex, with an orchestra of interacting molecular networks, each coming into play during precise stages of embryogenesis. An understanding of gene regulation throughout development is fundamental to establishing a more complete understanding of this process. Large-scale consortium projects, such as Eurexpress (1), have delivered near-comprehensive coverage of the transcriptome using *in situ* hybridization (ISH) to deliver gene expression profiles at key stages of embryo development. These data will assist in deciphering the gene expression networks that are driving the morphological changes associated with embryonic development. However, it is not until data are collated and organized into useable frameworks that their true potential for the elucidation of regulatory pathways involved in development is realized (2).

The EMAGE database aims to fully describe gene expression patterns within the developing mouse embryo. EMAGE is a freely available online database that includes spatially mapped patterns of mRNA ISH, protein immunohistochemistry (IHC) and transgenic reporter data (ISR) and serves as a means of organizing embryo image data generated by large screens such as Eurexpress. EMAGE is unique in providing both text-based descriptions of gene expression plus spatial maps of gene expression patterns. EMAGE uses 3D and 2D embryo models made publicly available by the e-Mouse Atlas Project (www.emouseatlas.org) as a spatial framework for mapping *in situ* expression patterns sourced from the literature, from mid- to large-scale screening projects and from direct submissions. Individual *in situ* expression patterns are mapped onto one of a series of Theiler (3) staged embryo models. Theiler staging criteria, which are based on morphological features, are used to stage-match between the source data and the embryo model. The spatial mapping technology utilized used by EMAGE, uses in-house developed image warping software with full-time editorial staff driving the mapping process and ensuring accuracy and consistency. It is the intention of EMAGE to develop 3D virtual models of *in situ* gene expression at successive stages of mouse embryo development, and it is the long-term goal of this project to deliver a 4D model of embryo development by which an end-user can explore trends in gene expression throughout development. Here, we review our progress in spatial mapping and data integration, and our recent developments to the EMAGE web interface to allow for enhanced query and analysis capabilities.

## 3D SPATIAL MAPPING OF OPTICAL PROJECTION TOMOGRAPHY DATA

Spatial mapping uses a set of virtual embryo models pertaining to each stage of post-implantation mouse development as categorized using Theiler’s system for stage definition (3). This spatial mapping of gene expression images to a standard model allows for comprehensive spatial annotation and ensures that data-rich image data are archived in a format that are amenable to subsequent computational search and analysis. Critically, it also paves the way for the implementation of tools developed to query across image data sets, and execute computational analysis of *in situ* (ISH, IHC, ISR) data. Recently, EMAGE has developed a method of spatially mapping 3D embryo images captured using optical projection tomography (OPT). OPT was developed to fill the so called imaging gap that fell between the high resolution optical microscopy capable of tissue penetration not beyond a few hundred micrometres, and the sub-tissue resolution scanning of techniques such as computed tomography and magnetic resonance imaging (4,5). This technology is ideally suited for specimens between 0.5 mm and 10 mm, making it practical for the imaging of mammalian embryos. Critically, OPT allows 3D imaging of whole mount embryos that have been assayed through colourimetric *in situ* protocols.

EMAGE uses an in-house developed graphical interface called WlzWarp to spatially map OPT data onto stage-matched models. While it is possible to use automated methods to spatially align high-contrast computed tomography (6) and magnetic resonance imaging (7) 3D embryo images, experiments using advanced neuroimaging tools (8) to spatially register OPT embryo data to a stage-matched model requires as much editorial correction as direct manual alignment, and as such is not practical for use in a spatial mapping pipeline (9). Spatial mapping using WlzWarp is a manual process and requires the placing of landmarks onto both source (3D image data) and target (virtual embryo model) with, on average, 100–200 landmarks needed for successful registration of embryo data. Once sufficient landmarks have been placed, a user can ‘warp’ the source data onto a 3D mesh that represents the virtual embryo model. The warping process itself uses a novel constrained distance transform (CDT) method to generate a spatial transform. CDT uses a radial basis function within a constraining geodesic mesh (10). The major advantage of the CDT method is that it is capable of 3D mapping embryo specimens that, although stage-matched by morphological criteria, can be highly variable in visual presentation. For example, the tail can curl to either the left or the right. A user of the WlzWarp interface can review the accuracy of the WlzWarp mapping process at any point through viewing the warped source data overlaid onto the target model, and can add additional landmark points as considered necessary.

Following spatial mapping, the warped data are segmented to define 3D domains of gene expression corresponding to the levels of expression (strong, moderate, weak, possible, not detected) that are accepted by the EMAGE database. EMAGE editors routinely use additional in-house developed imaging software, such as WlzViewer and MAPaint, to accomplish this task. To date, the EMAGE editorial office has carried out the spatial mapping of the 3D OPT patterns. However, it is important to note that all software applications that we have developed for this purpose are open source and free for the research community to use.

## VISUALIZATION AND QUERY OF 3D DATA

**Figure 1.**
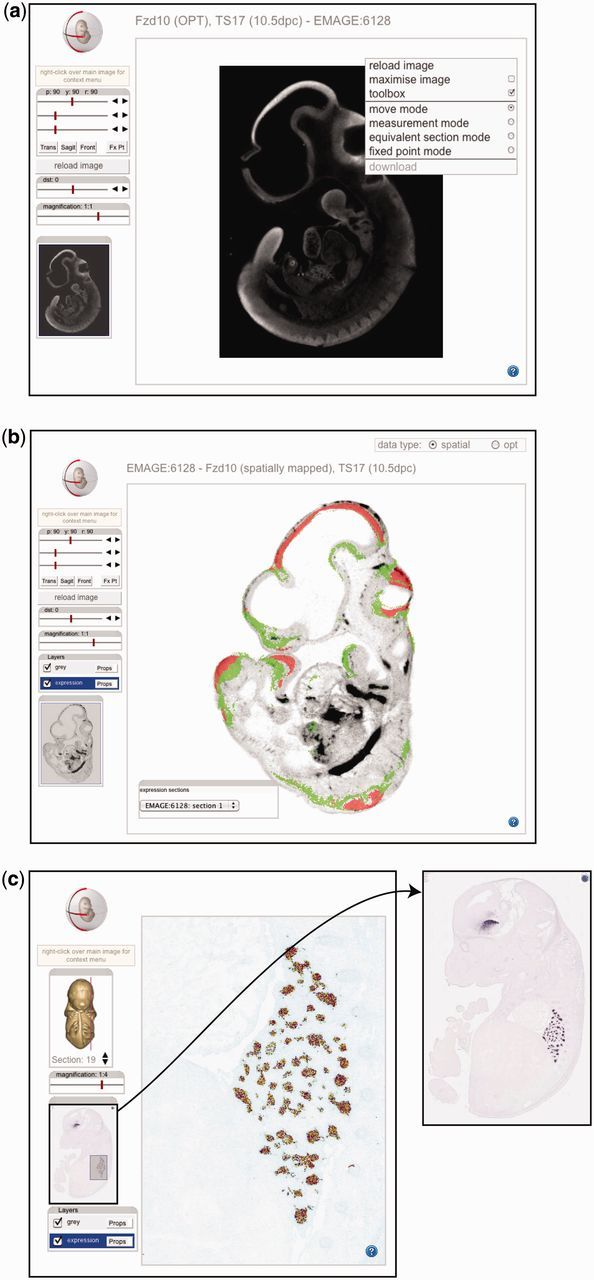
Use of IIP3D web browser-based section viewer in EMAGE (**a**) The IIP3D viewer allows arbitrary sections through OPT 3D data images to be viewed. The IIP3D viewer displays sections through large 3D objects (>100GB) by only downloading the pixels that you see. The advantage of this system is that large 3D images can be accessed using a modern web browser, such as recent versions of Firefox, Chrome or Safari, and software downloads are not required. A context menu offers additional options, including measurement mode that allows a user to measure the distance between two points. (**b**) Mapped OPT data can also be viewed using the IIP3D viewer. This image shows the data in panel (a) spatially mapped onto the appropriate EMAP model. The ‘expression detected’ domain is coloured red and ‘possible expression’ is coloured green. (**c**) Eurexpress section data. The tiling method of the IIP image server allows users to access the original serial section images within the context of a web browser. This obviates the need to download large image files. In the large central panel, an automated segmentation method has been used to generate coloured domains representing different levels of expression (red: strongest; yellow: moderate; green: possible; cyan: not detected). Section navigation is accomplished using the left panel. The original section images can be seen in the smaller panel to the right.

**Figure 2.**
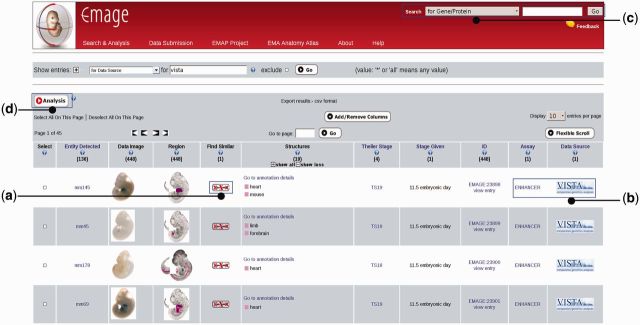
New and novel features of EMAGE. This figure shows a screen capture of an EMAGE results table with some new and novel features highlighted. (**a**) The ‘find similar function’ option retrieves a list of spatially similar EMAGE expression patterns, ranked by similarity, and can be accessed by clicking on the x-X-x icon. (**b**) EMAGE has implemented direct links to screens and resources whose data we represent. The enhancer data were obtained from the VISTA enhancer screen, and this is acknowledged in the data source column. (**c**) Quick search, which can be found on every page, allows users to select a category and perform a single criteria query. (**d**) The ‘Analysis’ option allows a user to export a gene list generated by EMAGE to ToppGene for extended analysis.

An IIP3D web tool (11) has recently been deployed to the EMAGE interface. This tool allows users to view arbitrary sections of large-volume 3D data using a web browser. The displayed image, which represents a section through a 3D volume, consists of rectangular tiles served by a WlzIIPServer (11). Pan-and-zoom capabilities allow the user to explore a chosen section, while a context menu offers further capabilities, such as the ability to measure the distance between two points on a section plane (Figure 1a). A toolbox provides navigation tools that allow the user to change section plane via translation (distance) or rotation (pitch, yaw). For ease-of-use, shortcuts are available that directly navigate to the cardinal planes through the 3D image (i.e. transverse, sagittal, frontal), as these are widely used by researchers in the field. In addition to allowing users to view arbitrary sections through original OPT image data, the EMAGE IIP3D tool provides a user-friendly interface to browse spatially mapped OPT and section data without the need to download large-volume 3D data sets (Figure 1a and b). In principle, this tool can be used to interactively view multiple gene expression patterns that have been spatially mapped onto a single virtual embryo model. Furthermore, we propose to develop the EMAGE IIP3D interface to allow spatially defined 3D queries to be performed across the EMAGE database. The latter will allow a user to choose either a section or sections of interest and ‘paint’ a query region of interest. This search by ‘embryo space’ capability is already in place for whole mount data (12) where it uses drawing tools to define a query region on a 2D whole mount-projection of an embryo model. It is our intention to use the EMAGE IIP3D technology to greatly extend this search ability to include user-defined arbitrary regions of 3D embryo models. Towards this end, it is important to recognize that 3D queries are already a feature of the EMAGE database and can be accessed using the ‘find similar’ function (Figure 2a). This function retrieves a list of spatially similar EMAGE expression patterns, ranked by similarity, and can be accessed by clicking on the icon marked [x-X-*x*], which can be found in both the results list (the ‘find similar’ column) and the EMAGE entry page. Briefly, the ‘find similar’ function works by comparing by Jaccard Index, all spatial patterns that have been annotated to the same model. A Jaccard index similarity coefficient score is generated for each pairing of patterns. A score of 1 indicates an exact spatial similarity between two mapped patterns, with progressively smaller scores indicating patterns that are progressively less spatially similar. These results are returned in a table of EMAGE entries ordered by spatial similarity to the original query pattern. Lists returned from any EMAGE query can be alternatively ordered by use of the column headers. For example, gene lists can be ordered alphabetically by clicking on the column header for ‘entity detected’. Although the ‘find similar’ described here enables users to compare between global gene expression patterns, we anticipate that the user-defined 3D query will be used to explore smaller regions of interest. As a consequence, they should be seen as complementary methods of finding 3D data.

## VISUALIZATION OF HIGH-RESOLUTION 2D DATA

The IIP viewer functionality described previously has also been extremely useful in the delivery of high-resolution 2D data within a web browser. EMAGE hosts a number of data sets, some of which represent large images of high-resolution section data. The Eurexpress data set (1) is one such example. Previously, delivering such large data-rich images within a web resource would have been slow and unwieldy for the user, and almost impossible without sufficient bandwidth. We have used the tiling method of the IIP image server to allow users to access these large original images within an IIP viewer (Figure 1c) quickly and easily, without the need to download large image files individually. In much the same way as with the 3D data, the user can scroll across the image, zoom in on regions of interest and toggle on and off the spatial mapping of the original data sections.

## COLLABORATIVE DATA CAPTURE

Originally, the image data acquired for mapping and inclusion in the EMAGE database were sourced from the literature in a bid to provide a large base of data on which to query. With this supporting base of spatial data readily available, more recently the focus has turned to collaborative data capture to provide complete subsets of data within EMAGE. These complete subsets could be of the form of an entire regulatory pathway represented at specific stages, or a comprehensive set of data based on type, e.g. transcription factors or regulatory enhancers. Described here is a sample of our current collaborative ventures to map mouse embryo image data of high biological impact.

## 2D SPATIAL MAPPING OF *IN SITU* IMAGE DATA FROM LARGE SCREENS

VISTA is a resource of experimentally validated human and mouse non-coding genomic DNA fragments with gene enhancer activity as assessed in transgenic mice (13). In this project, enhancer candidate sequences were identified by extreme evolutionary sequence conservation or by ChIP-seq. PCR primers were used to amplify conserved regions and ChIP-seq peaks, with the primers chosen extending by several hundred base pairs in both directions to include the flanking sequence required for enhancer activity. The PCR products were then cloned into an Hsp68 coupled LacZ reporter vector and microinjected into fertilized eggs. The embryos were harvested at 11.5 dpc, and stained for LacZ, and the resulting activity patterns annotated. EMAGE are currently spatially mapping these data (>1000 elements) for inclusion in the database, enabling spatial comparisons between enhancer activity and gene expression. The set of VISTA mouse non-coding fragments has now been successfully included in EMAGE as a new ‘enhancer’ data type, and queries have been developed that allow users to find these data easily. We are extending our query capabilities to include ‘search by chromosomal location’, and this will allow a user to find expression patterns of genes and enhancers within or near to a locus of interest. As part of our standard operating procedure, we have mapped the text annotation terms used by the VISTA screen to the EMAP ontology, and we have Theiler-staged every VISTA image that has been subsequently spatially mapped. The consequence of this is that we have developed a resource for investigating the spatial context of gene-enhancer relationships within a defined stage range (Theiler stage 16–19). We see this feature as being particularly useful to researchers who wish to identify colocalized putative enhancer elements and gene expression profiles.

## SPATIAL CROSS-INDEXING OF EMBRYS DATA

**Figure 3.**
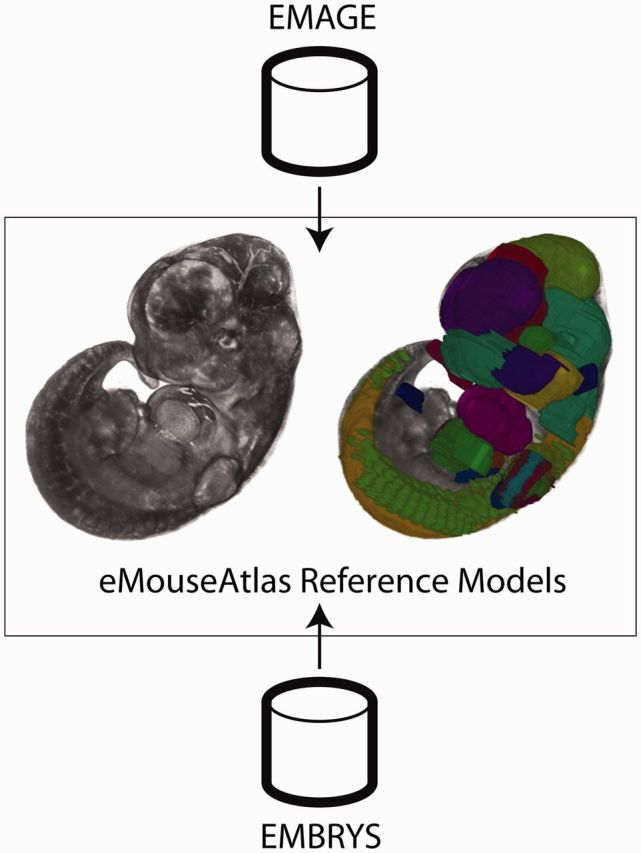
Using eMouseatlas to cross-index EMAGE and EMBRYS. The central panel shows the eMouseAtlas TS19 reference model (left) and additionally this model with multiple anatomical components delineated (right). Anatomical domains were prioritized for segmentation if they were included in the text annotation of the EMBRYS whole mount ISH screen. Segmenting these domains allows the eMouseAtlas framework to bridge between EMAGE (spatial- and text-annotation) and EMBRYS (text-annotation) database resources and allows EMAGE to develop inferred spatial annotation for EMBRYS gene expression patterns.

EMBRYS is a resource of ∼24 500 whole mount gene expression images (∼1.5 K genes) from 9.5 dpc, 10.5 dpc and 11.5 dpc mouse embryos (14). The images generated by the EMBRYS project detail the profiles of transcription factor and transcription factor-related factors in mouse development. In a collaborative effort aimed integrating the EMBRYS and EMAGE gene expression databases, we have created direct links between the resources. The developers of EMBRYS have implemented direct gene-by-gene links that allow EMBRYS users to find EMAGE data easily, and have re-structured their gene entry pages to include annotations to key anatomical components. To complement this, we generated segmented anatomical domains on TS15, TS17 and TS19 embryo models that reflect the key anatomical structures annotated by EMBRYS (Figure 3). These ‘anatograms’ allow us to develop ‘an inferred spatial annotation’, created automatically for each EMBRYS entry, and so will permit the EMAGE user to find EMBRYS data using a spatial query. The use of ‘inferred spatial annotation’ in this way enables spatial cross-indexing between EMAGE and EMBRYS, and allows users of EMAGE to explore spatial relationships between patterns that have been spatially mapped (e.g. OPT data, VISTA data) and a comprehensive set of transcription factors that have been text annotated at key stages of development.

## DATA INTEGRATION AND DISTRIBUTED SYSTEMS

### Integration of EMAGE with eMouseAtlas

**Figure 4.**
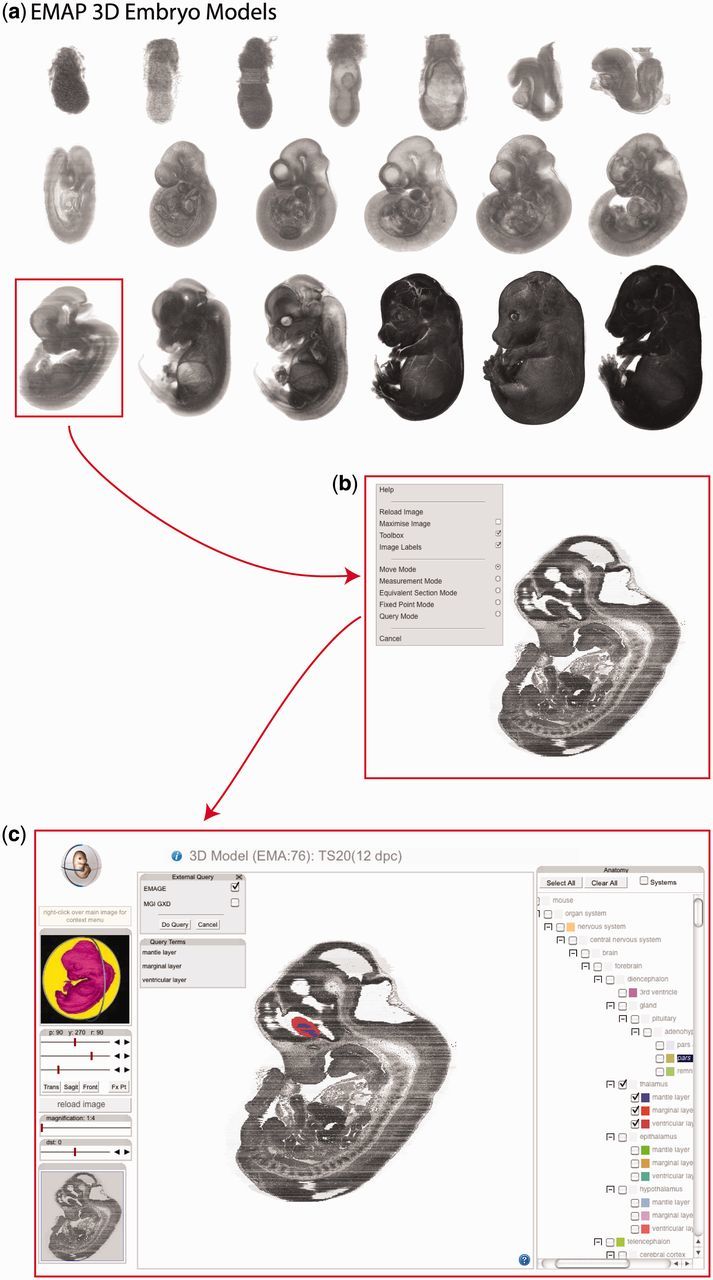
Querying EMAGE using eMouseAtlas. (**a**) eMouseAtlas (EMAP) provides 3D models of mouse embryos across development. These models are used as a framework to store spatial information such as delineated anatomical domains. (**b**) An IIP3D section viewer allows arbitrary sections through 3D EMAP models to be viewed. A context menu offers additional options, including query mode that allows a user to query EMAGE and GXD. (**c**) Multiple anatomical domains can be selected for an EMAGE query, and these appear as coloured domains in the section viewer. Section navigation is accomplished using the left panel. On the panel to the right, checkboxes allow users to toggle on/off the anatomical domains that are displayed in the section viewer.

EMAGE uses the atlas embryo models (Figure 4a) developed by eMouseAtlas as a spatial framework where gene and enhancer expression patterns extracted from raw data images can be spatially mapped. The atlas embryo models additionally allow users to identify anatomical structures through the provision of delineated anatomical structures on a subset of models. We have developed a means of integrating anatomy and gene expression so that a user of the atlas can query the EMAGE database. This feature uses the ‘query mode’ (Figure 4b) made available through the context menu of the IIP3D viewer to allow a user to select anatomical components of interest. Users have the option of using the selected anatomical domains to search either EMAGE or the MGI Gene Expression Database (GXD) (Figure 4c). Behind the scenes, this feature uses the IIP3D server to detect the EMAP ontology ID associated with each anatomical structure and encode this in a URL used to invoke the query. Both EMAGE and GXD have developed the EMAP ontology, which uses ‘part-of’ relationships to describe anatomical components throughout mouse development from the one-cell egg (TS01) through to the prenatal stage of development (TS26) (15). By virtue of using this shared ontology, it is relatively straightforward for eMouseAtlas to develop ‘point-and-click’ queries that can be performed across both gene expression databases.

We are extending this query to allow searches of user-defined arbitrary regions of atlas models. In essence, this is similar to the 3D query tool outlined earlier for the purpose of directly querying embryo space. In this instance, however, the objective is to develop an interactive 3D viewer that can simultaneously allow a user to browse delineated anatomical domains, perform database queries based on anatomy ontology (EMAP) IDs and perform spatial queries based on spatial domains in the EMAP atlas framework.

### Integration of EMAGE with KEGG

EMAGE has developed an integrated query with the Kyoto Encyclopedia of Genes and Genomes (KEGG) allowing users to use KEGG pathway descriptions as a means of querying the EMAGE database. This query uses web services to query the KEGG resource. As a means of organizing the returns from this query in a more structured and logical manner, we developed our ‘gene and pathway summaries’ view of EMAGE data. The gene summary feature orders images and text annotations in ‘gene strips’, whereby all EMAGE entries pertaining to a single gene at any one stage of development are condensed into a single row of a gene summary table. This alternative method of viewing data allows users to explore raw image data and text- and spatial-annotation structured through chronological sequence based on Theiler stage. By clicking on a thumbnail data image, a user can access the EMAGE entry associated with that particular thumbnail image. The pathway summary can be seen as a further development of the gene summary that uses gene lists provided by the KEGG database to deliver ‘pathway strips’ of EMAGE data. Similar to the gene summary, all EMAGE entries pertaining to a KEGG pathway description are condensed into a pathway summary table with each row relating to a developmental stage. There is further similarity to the gene summary table in that, by clicking on an image, the user is taken to the associated EMAGE entry where raw data images, probe details, text- and spatial-annotation and ancillary data can be accessed.

### Linking EMAGE to relevant resources

To fully use the potential of the data captured in EMAGE, it is desirable to create links between these data and other relevant resources wherever possible. This allows users to easily and logically navigate across and between resources, and thus glean the maximum information from all available sources. To this end, EMAGE has instigated a number of direct links to additional database resources, published articles and citation indexers. Every EMAGE entry has a series of links to GXD, Ensembl, Allen Brain Atlas, IKMP, BioGPS, EMBL-EBI and BrainStars. These links automatically perform a query at the remote resource for the mouse gene symbol specified by the EMAGE entry. In the case of resources dealing with organisms other than mouse, the link is to the orthologous gene for that organism. For example, EMAGE links to the GEISHA chicken ISH database (16). To find orthologous genes, EMAGE uses MGI vertebrate homology class, which invokes NCBI HomoloGene. HomoloGene programmatically detects homologues among the genome features of completely sequenced eukaryotic genomes. The input for HomoloGene processing consists of the protein sequences from the input organisms. These are compared with one another using blastp and are grouped using a tree built from sequence similarity to guide the process. Using HomoloGene, paralogs are identified by finding sequences that are closer within species than other species. Orthology allows the user to easily compare expression patterns across organisms, and thus to fit the gene expression information into an evolutionary context. In addition, for data sourced from *in situ* screens and/or other database resources, EMAGE now provides direct links to the original data source as a means of acknowledging where EMAGE obtained the image and text-annotation data (Figure 2b). These links can be accessed by clicking on the logo for the appropriate data source, which currently includes the following: EMAGE, EmbryoExpress, Eurexpress, FaceBase, MGI and VISTA.

More recently, EMAGE has developed reciprocal links with Elsevier articles. EMAGE includes significant data originally published in Elsevier journals through a copyright agreement allowing EMAGE to display the original images and, as such, has always included links back to the original journal article from within the EMAGE entry (as with all published data from the literature). Elsevier recently implemented reciprocal links for a variety of data repository resources, including EMAGE. This means that researchers reading an Elsevier article online will be able to see that data from that publication have been mapped and included in EMAGE. By clicking on the EMAGE button within the article, a query is performed on the EMAGE database for all EMAGE entries pertaining to that article, with the results being returned in a browser window. This allows the user a simplified method of browsing EMAGE entries, in the context of the journal article describing the original data. This functionality was made possible by the inclusion of DOI as a valid query string in the EMAGE formattable URL system.

One further relevant linking effort is the contribution to a citation index for data indexed by EMAGE. In collaboration with Thomson Reuters, EMAGE data can now be included in the h-index calculations of Web of Knowledge (http://thomsonreuters.com/web-of-knowledge/). This attributes credit for the data to the original author and to EMAGE, resulting in a measurable impact factor for EMAGE.

## QUERY AND ANALYSIS OF EMAGE DATA

EMAGE aims to generate a powerful and intuitive web interface that allows developmental biologists to ask important questions about embryonic development. Towards this end, we continually extend our data repository and our repertoire of query and analysis options available for EMAGE. Where possible, this is developed in consultation with our users in an effort to produce a web interface tailored to the needs of the end user. The nature of the spatial annotations held in EMAGE enables complex query and visualization solutions that are unique to this resource. In addition, it is our aim to ensure that even the novice user can access our data easily and quickly. In this respect, to allow navigation of the complex data available in EMAGE, we have implemented an advanced/combination query, allowing users to deliver bespoke queries across the EMAGE database. Furthermore, we have developed a BioMart interface that provides a common interface widely used by many database resources. The relative merits of each of these developments are discussed later in text. In addition, we report on the recent provision of analysis tools that are available from within the EMAGE interface.

### BioMart

EMAGE has implemented a BioMart interface where structured text queries can be performed on the EMAGE data set (17). BioMart provides a generic web query interface, and results can be viewed as a table within the web browser, or alternatively exported in HTML, CSV, TSV and XLS formats. In addition, the query can be exported in URL, XML or Perl formats. As a consequence of being able to export a query, it is extremely straightforward to repeat a query at a future time to determine whether, for example, additional patterns have been added to the EMAGE database. BioMart is extremely useful for advanced queries aimed at generating lists of EMAGE entries and lists of genes associated with text annotation of gene expression and/or other text-based details (e.g. author, specimen type, assay type). However, BioMart cannot provide spatial searches or direct access to image data, and for this functionality, we recommend using the EMAGE Combination Query.

### Combination query and quick search

The Combination Query is a powerful tool that enables users to create complex Boolean queries. Users can choose between an extensive list of categories, including some familiar options such as gene symbol, stage of development (i.e. Theiler stage), anatomical terms used in text annotation and functional annotation through the use of Gene Ontology (GO) terms. Additional categories include the following: specimen type, annotation type, data source, assay type, detection reagent, mutant allele, specimen strain and author. The strength of the Combination Query lies in the ability to add multiple criteria together to form a complex query. An expandable multi-input selector allows the user to select categories, and once a category is selected, predictive text assists the user in finding listed options. For categories with a relatively small list of options, a wildcard (*) allows a user to view all listed options. The default behaviour of the Combination Query is to deliver an OR query within levels (comma-separated list), an AND query between levels and the ‘exclude’ checkbox adjacent to each input being used to invoke a NOT query to the Boolean logic. These same categories available in the combination query can additionally be accessed via the improved version of the ‘Quick Search’ that is available on the EMAGE menu bar (Figure 2c). The new ‘Quick Search’ allows users to select a category and perform a single criteria query. A help button adjacent to the ‘Quick Search’ opens a pop-up window that lists the query terms that are enabled for each of the categories.

### Functional analysis of EMAGE data using ToppGene

A critical aspect of bioinformatics is the ability to bring data together for extended analysis and data mining. Significant results can be obtained using published data for novel analysis by linking data sets that are otherwise independent. The ToppGene Suite (http://toppgene.cchmc.org) addresses this need and allows biomedical researchers to perform gene list enrichment analysis and candidate gene prioritization based on functional annotations. ToppGene was developed using the open-source statistical language R (http://www.r-project.org/) and uses a fuzzy-based similarity measure to compute the similarity between any two genes based on semantic annotations (18). In an effort to integrate EMAGE and ToppGene, we have developed an EMAGE ‘analysis’ feature that allows a user to export a gene list generated by EMAGE to ToppGene for extended analysis. This ‘analysis’ feature (Figure 2d) can be accessed from the table view of EMAGE entries that is returned following a query by embryo space, combination, gene or anatomy name. On clicking the analysis button, a user is directed to a page where all genes from the EMAGE list are displayed. On this page, there are options to ‘show top 50 genes as EMAGE gene summary’ or to ‘analyse top 50 genes in ToppGene’. The latter option uses ToppGene to mine other database resources for functional annotation enrichments (phenotype, protein–protein interactions, GO annotation and so forth) of the gene list in question. This combined-resource analysis pipeline allows users of EMAGE to explore interactions and pathways critical for the development, function or dysfunction of specific organ systems in the mouse. Furthermore, through use of the EMAGE ‘embryo space’ query, this analysis can be limited to user-defined arbitrary regions of the developing mouse embryo. The latter query method is unique to EMAGE and highlights the importance of integration and interoperability between EMAGE and other resources.

### Direct access of EMAGE database

The principle method of accessing EMAGE data content is via the browser-based search and query functions (as described). However, it is also possible to use direct computational access to the underlying data by a number of methods.

Arguably the most comprehensive methods of computational access are by SQL query and by Java API. Because of the complex nature of the underlying data structure, we suggest that users wishing to use these access methods contact EMAGE for instruction. There are also computational access methods that do not require registration before use, such as query by Webservices, by DAS and by formattable URLs. EMAGE provides the service descriptions and documentation for web services on the EMAGE website to allow users to develop their own queries and analysis across the data set. Similarly there is a summary of the EMAGE DAS server commands provided, as well as a comprehensive description of the system for generating complex queries by URL. This ability to execute a query from the format of the URL makes it particularly easy to integrate EMAGE into tools and other resources.

### eMouseAtlas outreach

For a number of years now, EMAGE/eMouseAtlas has maintained a regular presence at key high-profile developmental biology conferences, through the presentation of a display exhibit within the conference exhibition. Specifically, EMAGE has regularly been exhibited at annual meetings of the British Society for Developmental Biology (BSDB) and the Society for Developmental Biology (SDB) as well as the quadquennial International Society for Developmental Biology Congress. Every year, EMAGE has also been exhibited at other relevant meetings likely to attract both current and potential new users. It is important to engage with users at every opportunity to determine the real-world requirements for the resource and to gather valuable feedback from our users, as well as the community in general. Through the provision of a display exhibit for the duration of the conference, it is possible to spend time speaking to users and offer one-to-one tutorials. This is additionally an opportunity to develop new collaborations with the Developmental Biology community aimed at focused data capture and spatial analysis of embryo data. Complementary to these face-to-face outreach efforts, EMAGE maintains a user-group/mailing list of users and interested parties, which facilitates announcements on new functionalities and data sets, while also acting as a source of potential contacts for feedback. Every EMAGE page also displays a feedback button, and we encourage our users to contact the resource at any time.

## CONCLUSION

EMAGE is a free online database resource for mouse developmental biology. It uses a series of 2D and 3D models of mouse embryos across development. These models are used as a framework to store spatial information, and we have used this strategy to collect spatially registered gene expression data. Through use of an IIP3D viewer, we have addressed the challenge of delivering 3D data via the 2D constraints of a web-browser. Furthermore, we have developed query and analysis methods that allow the research community to access this data easily using the EMAGE web interface. The true power of any bioinformatics resource is its integration with other resources, and towards this end we will continue to enhance the EMAGE database through direct links with other embryo and biomedical resources, through focused collaborative data capture and through the development of data analysis pipelines that make full use of spatial data.

## FUNDING

Funding for open access charge: Medical Research Council, UK, through core funding for the Mouse Atlas programme [grant number U.1275.2.4.4.1].

*Conflict of interest statement*. None declared.
